# Chloretone and Cocain in Combination for Extracting

**Published:** 1905-02-15

**Authors:** R. J. Winn

**Affiliations:** Bolivar, Mo.


### CHLORETONE AND COCAIN IN COMBINATION FOR EXTRACTING.

BY R. J. WINN, D.D.S., BOLIVAR, MO.

At the Jefferson City meeting of the Missouri State Dental Association, 1902, I had the pleasure of conducting a clinic, using cocain in combination with chloretone, since which time I have received so many letters of inquiry that I deem it expedient to write something with regard to my experience with said preparation, hoping thereby to benefit some one who is in trouble with the many operations we are called upon to perform which require a local anesthetic. I do not advocate the wholesale extraction of teeth, or pose as a "painless dentist," but experience in a country practice has taught me that a country dentist must be master of the situation and be able to do well the extracting that is demanded of him. The preparation which I use is cocain hydrochlorate, two percent, with chloretone, put up by Parke, Davis & Co. I am also using a one percent solution and find it very satisfactory when the gums are in a healthy condition, but in abscessed teeth and diseased gums the two percent solution is required. I claim for the preparation that a more thorough anesthesia is obtained, the effect is prolonged, a less amount is required, and, last but not least, the gums are left in an aseptic condition; the healing is hastened by the antiseptic properties of the chloretone in the solution. I use it in all operations where such things are required in a dental practice, but I wish to add that absolute care must be exercised if successful results are to be obtained. The hypodermic syringe must be in a perfectly aseptic condition, and one needle used but for one patient until it has been thoroughly sterilized. I do not think the idea of taking the solution from a wide-mouthed bottle a good one, but instead, it should be poured into a small dish of some kind. The needle should never be put into the bottle. I would not want to stand responsible for the results of unclean habits. The main point in using any local anesthetic is the manner in which the injection is made. I obtain best results by first injecting the medicine on the inside of the tooth about one-sixteenth inch up on the gums, right in line with the center of the root, then in the festoon between the teeth, a little on the inside. By using pressure with the finger the anesthetic effect can be forced to the outside so that there will be no pain when the outside is begun. In most cases the festoon is the only place the needle can be put on the labial side of the teeth. Care should always be taken to hold the end of the index finger on the soft tissue above the hard gum. It is most important that the medicine should be put in very slowly and the effect watched, and if one injection is enough to blanch the gums, stop when you have the gums white. In cases where several teeth are to be taken out after the needle is inserted the first time there need be no further pain, as the effect can be seen and injection made where the tissue is affected and following in that manner until the desired space of gum is covered. I operate in one and one-half to two minutes, but the effect is complete in thirty seconds, and lasts from five to twenty minutes, giving plenty of time to extract from five to ten teeth without being in any hurry. In case of scaling calculus from the teeth and particularly in pyorrhoea, I find this preparation very useful because of its lasting effect. I do not hesitate to use it for any patient, the result being very satisfactory. The syringe which I use is the one made especially for dentists by Parke, Davis & Co. From the cross bar (or finger hold) it has a long shank which fits in the palm of the hand which gives a much better lever power than if the thumb alone is used on the piston. I come in contact with many gums that it would be almost impossible to inject medicine into with an ordinary syringe such as physicians use. This dental syringe is fitted with adapter and is built for Schimmel's needles.—*The Dental Brief*, October, 1904.

